# Individual variation in preference behavior in sailfin fish refines the neurotranscriptomic pathway for mate preference

**DOI:** 10.1002/ece3.10323

**Published:** 2023-07-23

**Authors:** Rebecca L. Young, Sarah M. Price, Molly Schumer, Silu Wang, Molly E. Cummings

**Affiliations:** ^1^ Department of Integrative Biology University of Texas Austin Texas USA; ^2^ Department of Ecology and Evolutionary Biology Princeton University Princeton New Jersey USA; ^3^ Present address: Department of Biology Stanford University Stanford California USA; ^4^ Present address: Department of Integrative Biology University of California, Berkeley Berkeley California USA

**Keywords:** brain, mate choice, preference behavior, social affiliation, social context, transcriptome

## Abstract

Social interactions can drive distinct gene expression profiles which may vary by social context. Here we use female sailfin molly fish (*Poecilia latipinna*) to identify genomic profiles associated with preference behavior in distinct social contexts: male interactions (mate choice) versus female interactions (shoaling partner preference). We measured the behavior of 15 females interacting in a non‐contact environment with either two males or two females for 30 min followed by whole‐brain transcriptomic profiling by RNA sequencing. We profiled females that exhibited high levels of social affiliation and great variation in preference behavior to identify an order of magnitude more differentially expressed genes associated with behavioral variation than by differences in social context. Using a linear model (limma), we took advantage of the individual variation in preference behavior to identify unique gene sets that exhibited distinct correlational patterns of expression with preference behavior in each social context. By combining limma and weighted gene co‐expression network analyses (WGCNA) approaches we identified a refined set of 401 genes robustly associated with mate preference that is independent of shoaling partner preference or general social affiliation. While our refined gene set confirmed neural plasticity pathways involvement in moderating female preference behavior, we also identified a significant proportion of discovered that our preference‐associated genes were enriched for ‘immune system’ gene ontology categories. We hypothesize that the association between mate preference and transcriptomic immune function is driven by the less well‐known role of these genes in neural plasticity which is likely involved in higher‐order learning and processing during mate choice decisions.

## Graphical abstract

We identify neural gene expression patterns associated with female mate preference at the individual level in sailfin mollies. Our approach allows us to identify unique transcriptomic patterns associated with female preference behavior in a mating context distinct from other forms of social preference or social affiliation.

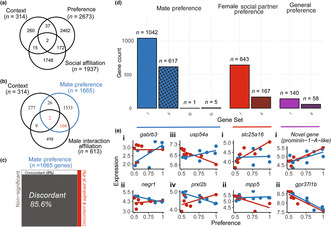

## 1 INTRODUCTION

Social behavior often mediates survival within populations as well as gene flow and reproductive isolation among populations. Thus, social behavior is crucial for ecological and evolutionary processes in natural populations; yet we are only just beginning to understand the underlying molecular mechanisms. Making decisions about whom to mate with is one of the most important social behaviors influencing fitness (Rosenthal, 2017; Rosenthal & Ryan, 2022), and it is one that we know relatively little about in terms of neurogenomic pathways (Cummings, 2015; DeAngelis & Hofmann, 2020; Ryan, 2021). The neural circuitry of mate choice is beginning to be well‐defined in a diversity of taxa as diverse as *Drosophila* (Clowney et al., 2015), rodents (Kavaliers & Choleris, 2017; Lenschow et al., 2022), and fish (Wong et al., 2012; Wong & Cummings, 2014; Yokoi et al., 2020). Yet, identifying the complex genomic pathways underlying these decisions is comparatively more coarse‐grained.

Research into the neurogenomics of female mate preference behavior has made great strides in identifying differentially expressed gene networks associated with females experiencing a mate choice event versus those experiencing a non‐mating context (Bloch et al., 2018, 2021; Cummings et al., 2008; McGraw et al., 2004), exposed to heterospecific versus conspecifics (Cui et al., 2017; Delclos et al., 2020), or selected for fast versus slow mating decisions (Mackay et al., 2005). These studies have used group‐level differences to identify candidate gene pathways that are engaged during a mate discrimination event such as those involving sensory processing (Bloch et al., 2018; Cui et al., 2017; Mackay et al., 2005) and neuroplasticity (Cummings et al., 2008; Delclos et al., 2020). Some of these candidate genes have been subsequently explored via manipulation (Ramsey et al., 2014; Wang et al., 2014) or examined for covariation with individual mate preference behavior (Lynch et al., 2012; Wang et al., 2014; Wong et al., 2012; Wong & Cummings, 2014). However, what is currently lacking in the field is an examination of neurogenomic networks that co‐vary with female mate preference at finer‐grained behavioral resolution (i.e., individual‐level) as well as differentiating mate preference pathways from other forms of social preference.

**FIGURE 1:**
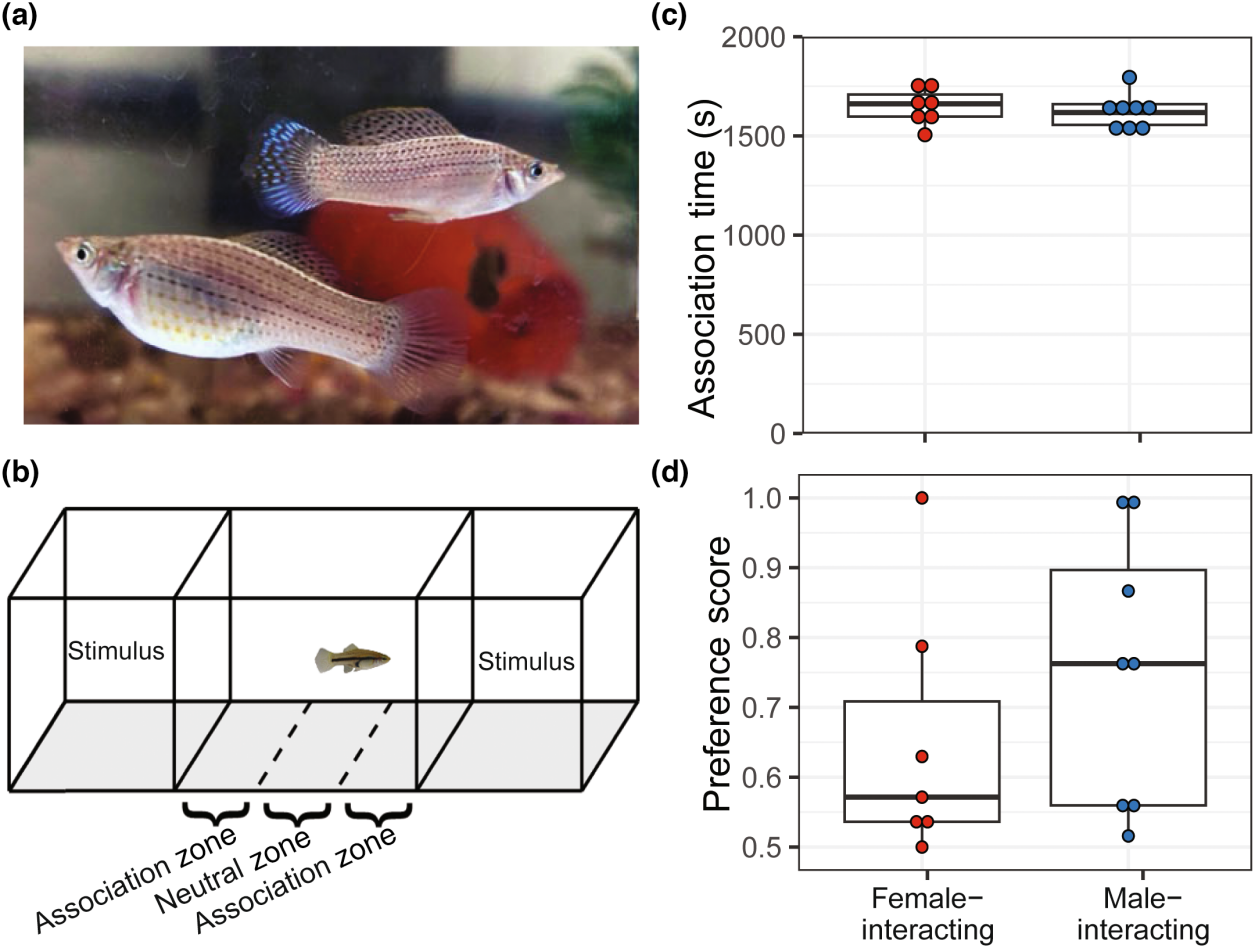
A female (large individual on the left) and male (small individual on the right) sailfin molly (*Poecilia latipinna*) in semi‐natural habitat (a). Schematic of the experimental setup (b) and individual and context‐dependent variation in social affiliation and preference behavior of focal females (c and d, respectively). Social affiliation was quantified as the total time (s) females spent in both association zones (24 cm in front of each stimuli compartment). Preference scores were defined as the proportion of stimulus‐directed movements towards one stimulus fish relative to the total number of stimulus‐directed movements towards both stimuli. While all animals included in the study exhibited high levels of social affiliation, affiliation did not differ between the female‐interaction context and male‐interaction context (c). Similarly, preference of focal females did not differ between the female‐ and male‐interacting contexts (d). Photo credit: Callen Inman.

Here, we move beyond characterizing group‐level variation in brain gene expression to examine transcriptomic profiles underlying individual variation in preference behavior. Our main objective is to determine if the brain genomic response for female mate preference is distinct from non‐mating social preference and whether it is differentiable from a more general social response (e.g., social affiliation). To elucidate the genes that are discretely expressed in mate choice, we evaluated brain gene expression patterns from female sailfin mollies (*Poecilia latipinna*, Lesueur, 1821; Figure 1a) that were making two different social preference decisions (mate preference or shoaling partner preference). We take advantage of the inter‐individual variation in female *P. latipinna* social behaviors to test the hypothesis that there are distinct neuro‐transcriptomic signatures underlying mate preference that differ from those involved in shoaling partner preference. We compare the brain transcriptomes from female subjects that varied greatly in social preferences (for either a mate and shoal partner) but not in time spent being social and then used a combination of approaches to dissect out the subset of genes that co‐vary with mate preference behavior distinct from other behaviors and preference in other contexts.

As behavior represents one of the most challenging phenotypes for neurogenomics studies (Fischer et al., 2021), we note that our approach advances the field in three ways. Firstly, this study represents the first attempt to identify transcriptomic covariation patterns with individual‐level variation in preference behavior. It is the differences in individual variation in behavior coupled with variation in neurogenomics that will elucidate more granular insight into the complex interaction between brain gene expression and behavioral response. Secondly, our individual‐level behavioral approach allows us to differentiate non‐target behaviors (e.g., social affiliation) from the behavior of interest (preference). Most group‐level approaches to behavioral genomics cannot segregate the contribution of the multitude of behaviors across individuals within a group to the aggregate brain genomic response. However, our individual‐level approach enables us to identify genes associated with preference behavior (i.e., behaviors biased towards one of two social options) from those associated with general social affiliation (i.e., time spent near conspecifics). Thirdly, by examining individual‐level preference variation in two different social contexts (mate choice and shoaling), we are incorporating both a non‐mating social control but also a behavioral control. We use size dimorphic stimuli in both our male‐interacting and female‐interacting contexts to elicit variation in preference behavior across social contexts. Preference for larger mating and shoaling partners is common across poeciliid fish (Agrillo et al., 2006; Dadda et al., 2005; Gabor, 1999; Marler & Ryan, 1997; Ptacek & Travis, 1997; Ryan & Wagner, 1987; Wong et al., 2011). By comparing neurogenomics across these two contexts, we can distinguish the gene pathways for mate preference from those involved in modulating shoaling partner preferences. Hence, this study represents the first attempt to identify gene sets that co‐vary with individual‐level variation in female mate preference behavior that is distinct from other forms of social preference. In total, these approaches provide a more fine‐grained lens to examine the underlying mechanisms regulating one of our most complex social decisions—mate choice.

## 2 MATERIALS AND METHODS

### 2.1 Behavioral trials and tissue collection

Sixteen adult female *P. latipinna* (Figure 1a) fish were collected from an on‐campus pond at the University of Texas, Austin during the breeding season from May to September 2012. All female subjects were >30 mm standard length (SL), had a visible brood patch (indicating they reach reproductively mature), and were therefore likely to represent adult females that had obtained significant experience (e.g., multiple months) under natural social environments with males. All animal collection, care, and experimental procedures were approved by the University of Texas at Austin IACUC (AUP‐2010‐00148, AUP‐2013‐00156).

We followed behavioral protocols from an earlier neurogenomics study (Cummings et al., 2008) with a northern swordtail (*Xiphophorus nigrensis*) that is also a member of the Poeciliidae family. Specifically, we took sexually experienced *P. latipinna* females from a semi‐natural environment, socially isolated them for 2 weeks to ensure sexual receptivity, subjected them to short‐term (30 min) non‐contact dichotomous choice assays, followed by immediate decapitation and brain removal for transcriptomic analyses. As in Cummings et al. (2008), we selected female subjects that exhibited high levels of social affiliation behavior (spending a minimum of 25 min in the association zones [see Figure 1b,c] along with a broad range of mate preference behavior [Figure 1d]) to maximize our ability to identify genes underlying individual variation in preference behavior while minimizing potential confounds (e.g., variation in social motivation). However, unlike the seminal swordtail study, we differed in the original protocols by (a) conducting individual‐level RNAseq analyses (rather than pooling brains within a treatment), (b) comparing only two social choice treatment groups (mate preference with male stimuli to female shoaling partner with female stimuli) without a non‐social control, and (c) having subject females make preference decisions based on stimuli size differences in both treatments. We specifically did not include a ‘non‐social assay’ as our initial swordtail study suggested females in this group exhibited significant behavioral (e.g., hyper‐activity) and neurogenomic stress responses. However, our shoaling partner (female‐interaction) treatment serves both as a non‐sexual social control, as well as a behavioral control. We specifically included size differences in both the male‐interaction and female‐interaction treatment groups to enable our ability to differentiate shoaling partner preference from mate preference using the same phenotypic axis. Female preferences for larger males as well as larger shoaling partner has been demonstrated across the poecilid family (Ryan & Wagner, 1987; Wong et al., 2011) and in sailfin mollies, in particular (Gabor, 1999; Ptacek & Travis, 1997). By having focal females make preference decisions along the same phenotypic axis (body size) but in different contexts (mating partner vs. shoaling partner) allows us to identify suites of genes underlying social preference responses associated with specific contexts.

In brief, our behavioral protocol involved randomly assigning subject females to either a female‐interaction (Ff) or male‐interaction (Mm) treatment group in which focal females would participate in a dichotomous choice assay to evaluate preference for either a shoaling or mating partner, respectively. Focal females in both treatment groups were similar in size ranging from 30.3 to 53.1 mm SL in the Ff group, and 32.3 to 51.4 mm SL in the Mm group, with no statistical difference in size between the treatment groups ((Mm): *n*_Mm_ = 8, Median (IQR) = 42.55 mm (11.30 mm) and (Ff): *n*_Ff_ = 7, Mdn (IQR) = 43.90 mm (11.15 mm); Mann–Whitney *U* (MWU), *U* = 29.5, *p* = .91; Young et al., 2023b: Table S1). After 2 weeks of social isolation, individual focal females were placed in the dichotomous choice experimental tank (see Figure 1b) consisting of five 24 cm × 30 cm zones: two end‐zones (occupied by stimulus fish) compartmentalized using Plexiglas dividers, two association zones (adjacent to the end‐zones), and a central neutral zone (between the two association zones that included a plant for cover). Prior to the introduction of the focal females, the pair of stimulus fish (a large and small male in Mm; or a large and small female in Ff) were acclimatized for 15 min in their respective end‐zones. The live stimuli differed in size in both treatments. Mm treatment stimuli paired a large male (54.6 mm SL) employing a courtship mating tactic with a small male (31.1 mm SL) employing a coercive mating tactic. Ff treatment stimuli paired a large 56.5 mm SL female with a small 40.3 mm SL female. Both the large male and female stimulus fish were larger than any of the focal fish (Young et al., 2023b: Table S1). Two focal females from the female‐interacting context were smaller (30.3 and 34.5 mm) than the small stimulus females (40.3 mm). The small stimulus male was smaller than all of the focal females tested in the male interaction context (Young et al., 2023b: Table S1). While repeat use of stimuli for behavioral experiments has its drawbacks for some experiments (e.g., pseudoreplicaton), it was of minimal concern here as we were using these stimuli to elicit a previously documented poecilid size preference (e.g., as in studies that use animated stimuli, see Reding & Cummings, 2017). As our objective was to finely characterize preference responses in the brain (not identify the traits females prefer), the specific attributes of the stimuli were less important than the range of preference responses they evoked in the subjects.

Each focal female was acclimatized in an opaque cylinder for 5 min in the central neutral zone before being released. Following release from habituation, focal females were observed for 15 min and position and behavior in the tank were recorded. After the first 15 min of observations, focal females were brought back to the neutral area and placed in the opaque habituation cylinder while the stimulus fish were switched to opposite sides to ensure that our preference measures were directed at a specific fish rather than a location preference in our experimental tank. Following the stimulus switch, focal females were observed for an additional 15 min. Immediately following the 30‐min behavioral trial, focal females were immediately decapitated, whole brains were removed and individually immersed in RNAlater solution (Applied Biosystems) at 4°C with gentle shaking for 24 h, then stored at −80°C (without RNAlater) for subsequent RNA profiling.

For all trials, we recorded social affiliation (time spent in both association zones, see Figure 1b), as well as preference behavior (biased behavioral movements directed towards one stimulus over the other). To characterize an ‘active’ preference for mating (in Mm) or shoaling (in Ff) partner, we recorded all movements directed at the stimulus fish: the frequency of up‐down movements (moving vertically along the Plexiglas dividers), back‐forth movements (moving horizontally along the Plexiglas dividers) and a glide‐like movement, i.e., a receptivity behavior as described in *X. nigrensis* (Cummings et al., 2008). We calculated a ‘preference score’ as the proportion of stimulus‐directed movements towards stimulus *a* relative to the total stimulus‐directed movements towards both stimuli, where stimulus‐directed movement towards stimulus *a* is greater than stimulus‐directed movement towards stimulus *b*. Therefore, our preference score varies from 50% (no preference or bias) to 100% (all movements directed toward one of the two stimuli in the tank).

### 2.2 Behavioral statistics

Statistical differences between the female‐interaction (Ff) and male‐interaction (Mm) social treatment for behaviors were assessed using the Mann–Whitney *U* (MWU) for non‐parametric distributions (Wilcox. test). Size preference (i.e., preference for the large stimulus vs. preference for the small stimulus) was evaluated using the binomial sign test (binom.test). To test the relationship between preference score and total affiliation time, we performed Pearson's correlation (cor.test). Behavioral statistics were performed using base R (version 3.6.1).

### 2.3 RNA extraction and library preparation

Total RNA was extracted from whole brain tissue using the Qiagen RNeasy kit following the manufacturer's instructions and quantified and assessed for purity on a Nanodrop 1000 (Thermo Scientific). Libraries for sequencing were prepared using Illumina's TruSeq mRNA Sample Prep Kit with minor modifications. Briefly, 1–4 μg of total RNA was used as input; mRNA was separated from total RNA using bead purification. After cDNA synthesis and chemical fragmentation, unique indices were ligated to each sample to allow for multiplexing. Libraries were amplified for 18 cycles and size distribution was verified using on the Bioanalyzer 2100 (Agilent) and single‐end sequenced on two lanes at the Lewis‐Sigler Institute for Integrative Genomics.

Libraries were demultiplexed using a custom python script. Raw 101 bp reads were trimmed to remove low‐quality base pairs (Phred quality score <20), reads with few contiguous high‐quality base pairs (fewer than 30 bp) using a python script (http://genomics‐pubs.princeton.edu/prv/resources/scripts/TQSfastq.py), and adapter sequences using the program cutadapt (https://cutadapt.readthedocs.io/en/stable/). To evaluate the quality of the trimmed reads, we ran FastQC (Andrew, 2010) using TACC (Texas Advanced Computing, The University of Texas at Austin).

### 2.4 RNA sequencing read quality evaluation, alignment, and quantification

After assessing the quality of the trimmed reads (using FastQC version 0.11.5), trimmed reads were pseudoaligned with Kallisto (version 0.43.0) using the single‐end reads option with a length of 100 bp (‐l 100), standard deviation of 20 (‐s 20). Transcripts were mapped to cDNA from two species retrieved from the Ensembl Genome Brower (Zerbino et al., 2018), the Japanese medaka HdrRr (assembly GCA_002234675.1, retrieved January 2019) and the Sailfin molly *Poecilia latipinna* (assembly GCA_001443285.1, retrieved January 2019). Ensembl transcript IDs, transcript counts, and transcripts per million (TPM) for all fish were consolidated into a counts file and a TPM file, respectively. Ensembl transcript IDs were converted to gene IDs (using biomaRt, Durinck et al., 2005, 2009). Because genes can have multiple transcripts, counts of transcripts from the same gene were summed (subsequently called ‘gene counts’). The use of the medaka reference resulted in fewer mapped transcripts and subsequently removed from further analysis. Sailfin‐mapped genes with more than two TPM in three or more individuals were retained. We used these remaining sailfin‐mapped genes (a total of 18,340 genes) for all subsequent analyses. Unless otherwise specified, all analyses were performed in R.

### 2.5 Gene expression patterns and association with social context and preference behavior

To identify genes associated with our behavioral measures (preference score or social affiliation), we used a linear model‐based approach (*limma*). We used *limma* (R package) (Ritchie et al., 2015) to identify distinct sets of genes: (i) genes that were differentially expressed (DEGs) between social contexts (male‐interaction and female‐interaction), (ii) DEGs that were correlated with preference behavior in each context (along with the specific direction of those correlations), and (iii) DEGs that were correlated with social affiliation in each context. Importantly, we compared the two behaviorally‐associated gene sets to distinguish genes associated with preference from those associated with a more general social response (e.g., social affiliation). Voom‐transformed gene counts were used as the input for *limma*. Briefly, voom performs log_2_‐transformation on the gene counts and uses a mean–variance relationship to produce a precision weight for each gene (Law et al., 2014). We used the *limma* approach to identify genes that have a slope significantly different from 0 and identified the specific direction of the correlation to determine genes that were positively correlated in both contexts or negatively correlated in both contexts (e.g., concordant expression) as well as those showing different correlation patterns across contexts (e.g., discordant expression). Raw *p*‐values are adjusted for multiple hypothesis testing using empirical false discovery rate (eFDR) (Storey & Tibshirani, 2003; after Lee et al., 2022). eFDR is a permutation‐based approach. Each iteration (*n* = 1000 iterations), treatment assignment (male or female choice context), and behavioral scores (preference and social affiliation) are randomly shuffled across the samples and limma‐based differential expression analyses and expression‐behavior association tests are performed on the randomized data to obtain a null distribution of *t*‐statistics to estimate empirical false discovery rate (*q*‐value) for each gene. Genes differing between contexts at an unadjusted *p*‐value < .05 and an eFDR *q*‐value < 0.05 were considered differentially expressed or associated with our focal behaviors for all downstream analyses.

To identify genes differentially expressed between the social contexts, we analyzed the difference in expression between the contexts (female‐interaction social context and the male‐interaction social context) using the model: ~0 + Context. To identify the genes associated with preference in each of the social contexts, we used the *limma* model: ~ 0 + Context + Context:Preference. We separated these results to identify ‘mate preference’ genes, i.e., those that correlated with preference in the male‐interaction context but exhibited no correlation or discordant correlations in the female‐interaction context. We identified ‘shoaling partner preference’ genes as genes uniquely correlated with preference in the female‐interaction social context but exhibiting no correlation with preference behavior in the male‐interaction treatment. To determine if any of these correlated ‘preference’ gene sets were distinct from those associated with social affiliation, we identified genes that are associated with social affiliation in the male‐interaction context with the *limma* model: ~ 0 + Context + Context:Social Affiliation and then examined gene list for overlap and directional concordance of the correlation with preference and social affiliation with males.

### 2.6 Gene co‐expression patterns and association with social context and preference behavior

To capture genes with coordinated expression variation across individuals, we performed a Weighted Gene Co‐expression Network Analysis (WGCNA) (Langfelder & Horvath, 2008). Voom‐transformed gene counts were also used as input for WGCNA. Using 9 as the soft‐thresholding power, genes were grouped based on their sign (i.e., positively correlated genes are grouped separately from negatively correlated genes; Langfelder & Horvath, 2008). Networks were constructed by grouping all 18,340 genes into a single block, merging correlated modules (correlation height >0.75), and using a minimum module size of 100. WGCNA clusters genes by expression similarity and summarizes gene co‐expression as module eigengenes ME (i.e., the first principal component of all the genes in each co‐expression module). Thus, each ME is the linear combination of gene expression values of all genes contained in the module that explains the most variation in the expression levels of the genes contained in the module.

We used linear models to assess the relationship between WGCNA gene co‐expression module eigengenes (MEs) and preference across contexts. Specifically, for each gene co‐expression module, we determined significance of preference behavior and social context on the ME (i.e., ‘ME ~ Context*Preference’). When the interaction model is not significant, we evaluated simpler models of the main effects of social context and preference independently. Model significance was determined after adjusting *p*‐values using the Benjamini‐Hochberg procedure, with an adjusted *p*‐value of *p* < .05 as the cutoff threshold. The ‘gray’ module (i.e., the module that groups unclustered genes) was excluded from these downstream analyses.

To identify a refined set of candidate mate preference genes, we combined the independent *limma* gene expression and WGCNA co‐expression analyses. Specifically, we compared the gene sets that were significantly associated with preference in a mating context at *p*‐value < .05 and eFDR *q*‐value < 0.05 with the WGCNA modules significant for preference. We identified genes associated with the WGCNA preference modules we selected genes exhibiting high ‘module membership’ (i.e., >|0.8|) with preference‐associated modules (after Hilliard et al., 2012). Module membership is calculated as the correlation of gene expression to the module co‐expression eigengene.

### 2.7 GO term enrichment in mate preference‐associated genes

To determine the functional gene ontology categories for the genes associated with (i) mate preference (identified with *limma*) and (ii) the refined mate preference genes (combining *limma* and WGCNA), we ran g:Profiler (g:GOSt) (Raudvere et al., 2019) using the ensembl *P. latipinna* (version P_latipinna‐1.0) Gene Ontology (GO) datasources. Genes from (i) and (ii) were separately used as input for the g:Profiler website, selecting the ranked‐based approach. Significant GO terms (adjusted *p* < .05) were computed using the multiple hypothesis testing corrections (g:SCS) that accounts for the hierarchical structure of GO terms (Reimand et al., 2007). A summary score indicating the overall direction of association of genes in enriched GO terms was calculated using a ‘*z*‐score’ where *z* is equal to (# of GO term genes with a positive association with preference—# GO term genes with a negative association with preference)/square root of the total # of term‐associated genes (after Walter et al., 2015). Significant GO terms were grouped by generalized associated process/phenotype.

## 3 RESULTS

### 3.1 Behavior

Fifteen of our 16 female subjects met our high social affiliation criterion (>25 min in association zones) and were included in the subsequent analyses. We removed one female that did not meet the 25 min total association time threshold from further analysis. By only including females with high levels of social affiliation that also represented a wide range of preference behavior (see Figure 1), we were able to focus on identifying gene sets that uniquely co‐varied with female preference behavior and not confounded by additional variance in social affiliation. These 15 females showed no difference in social affiliation between the two treatment groups (Figure 1b, Young et al., 2023b: Table S1, (*n*_Mm_ = 8, Mdn (IQR) = 1617.5 s (104.5); *n*_Ff_ = 7, Mdn (IQR) = 1662.0 s (111.5)); MWU, *U* = 33, *p* = .61, Cohen's *D* = 0.32). While females in both treatment groups exhibited high inter‐individual variation in preference, we observed similar range of preference behavior (biased movements directed towards one stimulus over another) in both mate choice and shoaling partner contexts (Figure 1c, Young et al., 2023b: Table S1, (*n*_Mm_ = 8, Mdn (IQR) = 0.76 (0.34); *n*_Ff_ = 7, Mdn (IQR) = 0.57 (0.17)), MWU: *U* = 20.5, *p* = .42, Cohen's *D* = 0.53). Mirroring results from other poecilid species, nearly all focal subjects exhibited a greater preference (i.e., biased movements) towards the larger stimulus in both contexts (*n*_Mm_ = 7 of 8 and *n*_Ff_ = 6 of 7 with 1 female having equal preference; binomial sign test *p* = .0074). Total association time was not correlated with preference score across treatments (Pearson's correlation: *r* (13) = −.25, *p* = .36).

### 3.2 Genes associated with social context and preference

#### 3.2.1 Social context genes (interacting with male stimuli vs. interacting with female stimuli)

**FIGURE 2:**
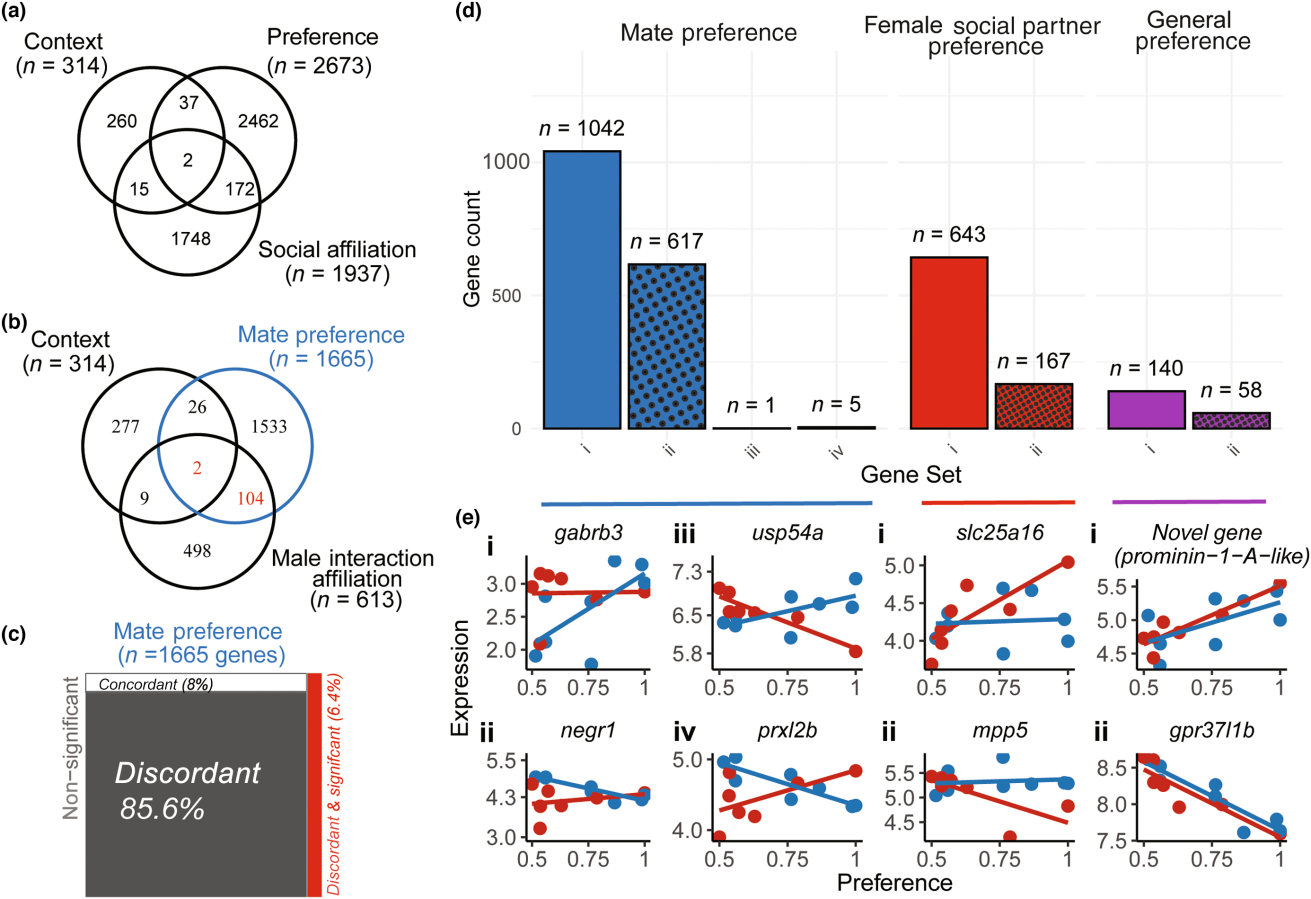
Genes correlated with preference and affiliative behavior in different contexts. (a) Venn diagram indicating the number of genes differentially expressed between the two social contexts (Context), genes correlated with social affiliation in one or more social contexts (Social Affiliation), and genes associated with preference in one or more social contexts (Preference). Genes are largely discrete between these broad categories with relatively few genes shared between each category. (b) Venn diagram indicating the number of genes associated with Male Interaction Affiliation, Mate Preference, and differentially expressed between contexts. (c) Treemap plot indicating that the 1665 preference‐related genes were largely discordantly expressed across preference and affiliative contexts. Red genes B and C were significant in both male preference and male affiliation; however, all of these genes exhibited discordant correlations with mate preference and male social affiliation. (d) Mate Preference genes (blue; i.e., genes uniquely or differentially correlated with preference in a male‐interacting context) were further categorized into four groups including: positively correlated (i), negatively correlated (ii, dotted box), expressed in opposing directions between male‐ and female‐interacting contexts (i.e., positively correlated with preference in the male‐interaction but negatively correlated in the female‐interaction (iii), or negatively correlated with preference in the male‐interaction but positively correlated in the female‐interaction (iv)). Preference genes associated solely in the female‐interaction context (red, Female Social Partner preference) were characterized as either positively correlated (i), or negative correlated (ii, dotted box) with expression. General Preference genes (purple) showed concordant expression with preference in both contexts, positive (i), or negative (ii, dotted boxes). (e) Individual expression of representative genes for each correlation described above are shown. Plotted are the expression of each gene on preference behavior from each focal female. Red and blue lines indicate the direction of expression between preference behavior and gene expression in the respective context and are not meant to represent a correlation. Names, statistics, and categories of each Mate Preference gene are provided in Table S3.

Of the 18,340 genes expressed in the brains of the 15 focal females following the 30‐min behavioral trials, we found 314 genes that were differentially expressed between the social contexts (Figure 2a; Young et al., 2023b: Table S2). One hundred and seventy‐five genes exhibited increased expression in the female‐interaction social context and 139 genes were increased in the male‐interaction context. Importantly, we found an order of magnitude more genes associated with behavior than social context alone (Young et al., 2023b: Table S2). Our *limma* analyses identified a total of 2673 genes associated with variation in preference behavior and 1937 genes associated with variation in affiliation behavior across the two social contexts (at *p*‐value < .05; eFDR *q*‐value < 0.05; Young et al., 2023b: Table S3). Genes associated with preference behavior exhibited very little overlap with genes associated with social affiliation (total association time). Only 174 of 2673 (6.5%) of preference‐associated genes were also associated with social affiliation (Figure 2a; Young et al., 2023b: Table S2).

#### 3.2.2 Preference genes: differentiating ‘mate preference’ from ‘shoaling partner preference’

Our *limma* analysis identified a total of 2673 genes associated with variation in preference behavior in the two social contexts (*p*‐value < .05 and eFDR *q*‐value < 0.05; Young et al., 2023b: Table S3). One thousand six hundred and sixty‐five genes (62.3%) were associated with mate preference (i.e., in the male interaction context, Figure 2d blue bars; and also Figure 2e for representative expression patterns). This set included all genes whose expression pattern did not correlate with preference behavior in the same direction as the genes associated with preference in the female‐interacting context (Figure 2d, red). We further characterized these genes by how they were associated with preference behavior across the two contexts. One thousand and forty‐two genes were positively associated with preference in the male‐interaction context while showing no significant association with preference in the female‐interaction context (Figure 2d, blue (i)). A representative of one of the genes positively associated with mate preference is the GABA_A_ receptor subunit (*gabrb3*; Figure 2e(i)). Notable genes involved in synaptic plasticity were found in this group (Figure 2d(i)) such as *ntrk2b* (a teleost *bdnf* receptor; Sahu et al., 2019), and a synaptogenesis gene similar in function to *neuroligin3*, *ptprsa* (receptor‐type tyrosine‐protein phosphatase S‐like). A total of 617 genes were negatively associated with preference in the male‐interaction context while exhibiting no association with preference in the female‐interaction context (Figure 2c(ii), blue) including neural growth regulator (*negr1*; Figure 2e(ii)). Additional genes in this category are the genes associated with neuroplasticity via neurogenesis (*s1pr1*, sphingosine‐1‐phosphate receptor 1; Guo et al., 2013) and via long‐term potentiation and NMDA receptor‐mediated signaling (*serpine2*, serine protease inhibitor; Kvajo et al., 2004; Lüthi et al., 1997). A much smaller subset of genes showed opposing patterns of expression patterns with preference behavior between the two contexts. USP54‐like (Figure 2e(iii)) was the only gene that positively associated with preference in the male‐interaction context and negatively associated with preference in the female‐interaction context (Figure 2d, blue (iii)). Only five genes were negatively associated with preference behavior in the male‐interaction context and positively associated with preference behavior in the female‐interaction context (Figure 2d, blue (iv)).

Our experimental approach further amplified our ability to isolate genes specific to preference behavior as we selected subjects with high affiliation (all females spent >25 min in the social interaction zone during the 30 min trials, see Figure 1b,c) but varied greatly in preference behavior (Figure 1d). Despite having reduced variation in social affiliation behavior across subjects, our *limma* social affiliation model analyses identified some genes with strong associations with social affiliation across taxa (e.g., *avp*: oxytocin/neurophysin I prepropeptide, see Young et al., 2023b: Table S2) providing validation for our approach.

To determine whether mate preference genes were similarly expressed during affiliative behavior in a male interaction context, we determined the directional concordance of the 1665 mate preference genes in a male social affiliation context. We found 0 genes significantly and concordantly associated with both mate preference and affiliation in a male interaction context, 106 genes (6.4%) significantly associated with mate preference and affiliation in a male interaction context in the discordant direction (e.g., positively correlated with preference and negatively correlated with affiliation), 133 genes (8.0%) concordantly associated with mate preference and affiliation in a male interaction context but not significantly associated with affiliation, and 1426 genes (85.6%) discordantly associated with mate preference and affiliation in a male interaction context and not significantly associated with affiliation (Figure 2b,c). Because all 106 genes significantly associated with both mate preference and social affiliation were discordantly expressed (Young et al., 2023b: Table S3, italicized), we did not exclude these genes as mate preference genes from downstream analyses.

Of the 2673 total preference genes, 810 genes (30.3%) were associated with female‐interaction preference (red boxes, Figure 2d; Young et al., 2023b: Table S3). A total of 643 genes were positively associated with preference behavior in the female‐interaction context while showing no association with preference behavior in the male‐interaction context (Figure 2d, red (i)) including *slc25a16* (solute carrier family 25 member 16; Figure 2e(i)) gene and *ipo11* (importin 11). One hundred and sixty‐seven genes were negatively associated with preference behavior in the female‐interaction context with no association with preference behavior in the male‐interaction context (Figure 2d, red (ii), see *mpp5* in Figure 2e(ii)). A total of 198 genes were concordantly expressed in both social contexts (Figure 2d, purple). Of these genes, 140 were positively associated with preference in both the male‐interaction and female‐interaction contexts (Figure 2d, purple (i); see prominin‐1‐A‐like in Figure 2e(i)). A notable gene in this category includes *Infgr1‐like* (interferon gamma receptor 1‐like). Fifty‐eight genes were negatively associated with preference behavior in both the male‐interaction and female‐interaction contexts (Figure 2d(ii), see prosaposin receptor, i.e., *gpr37l1b*, in Figure 2e(ii)).

### 3.3 Functional gene ontology categories for mate preference

**FIGURE 3:**
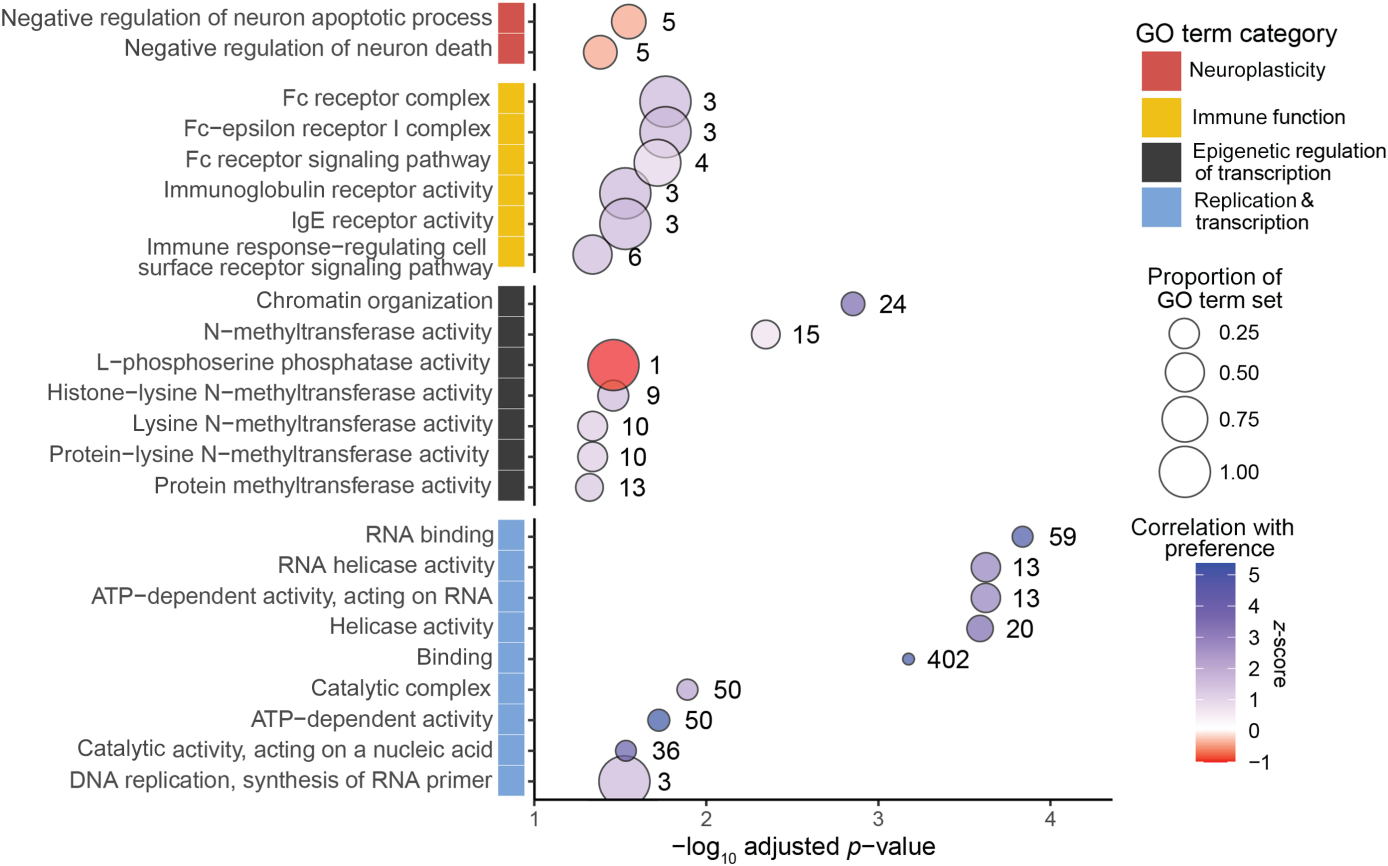
Gene Ontology (GO) terms significantly enriched (*p* adj < .05) in the Mate Preference genes (Figure 2d, blue). The number of genes per GO term is represented as the size of each bubble with the exact number flanking each to the right. Color of the bubble summarizes the overall correlation of the GO term genes with preference in a male‐interactive context with blue indicating a bias towards positive correlation and red a negative correlation. GO terms are grouped by broad functional categories including those associated with neuroplasticity, immune function, epigenetic regulation of transcription, and replication and transcription. GO term accessions, descriptions, and associated statistics are provided in Table S4.

To identify the enrichment of functional categories in our mate preference genes, we performed GO analysis on the 1665 mate preference genes identified through *limma* (Figure 2d, blue bars). We found 24 GO terms significantly enriched in this gene set (adjusted *p*‐value < .05). The 24 GO terms are broadly associated with neuroplasticity, immune function, epigenetic regulation of transcription, and replication and transcription (Figure 3). For each GO term, we characterized overall expression correlation with preference as GO term accessions, sub‐ontologies, enrichment statistics, and associated genes (Young et al., 2023b: Table S4).

### 3.4 Co‐expression networks correlate with preference behavior

**FIGURE 4:**
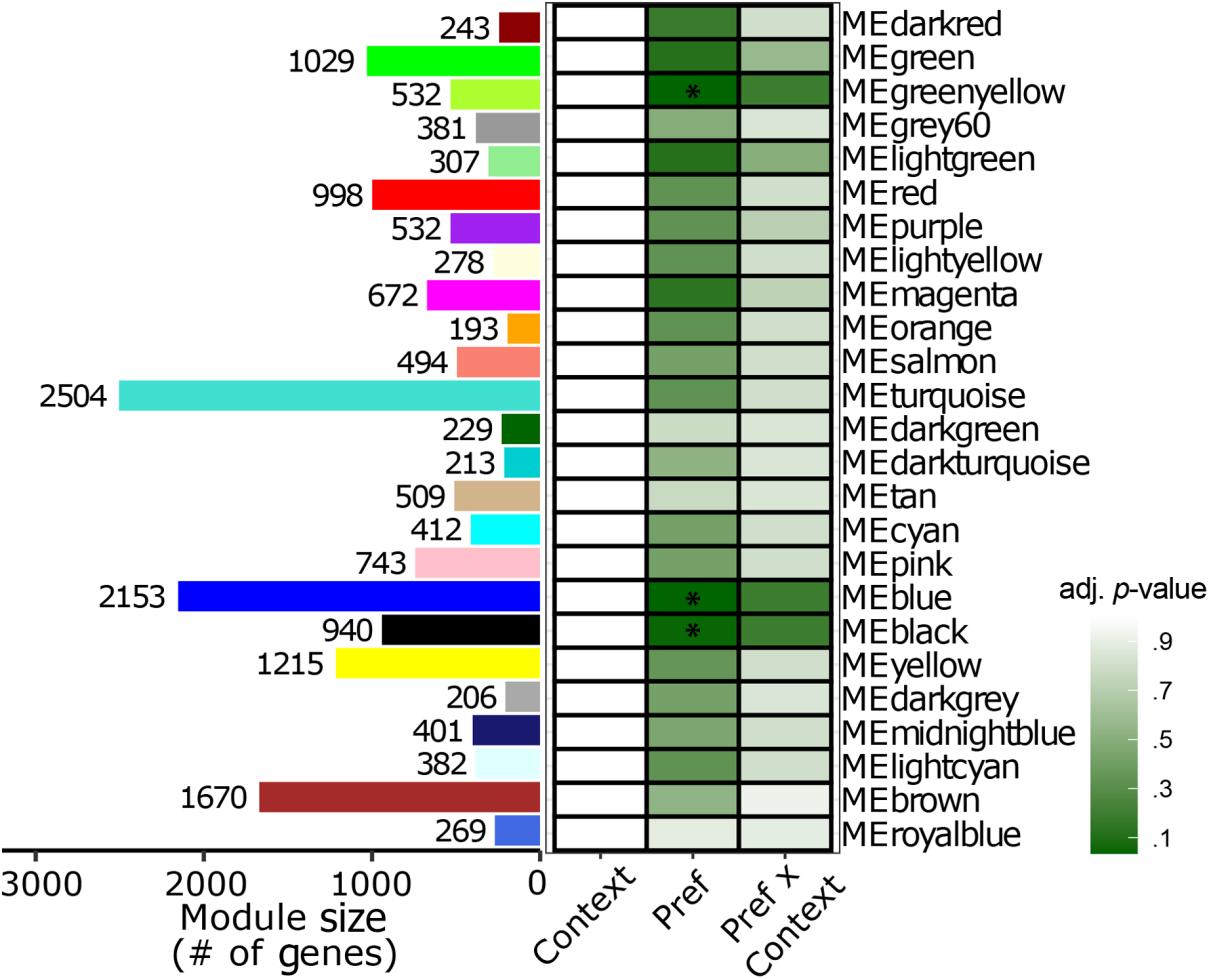
Modules of 25 co‐expressed genes (WGCNA) are reported as number of genes per module (represented as color bars on the left). For each module, the difference in eigengene expression between male‐interacting and female‐interacting context, the relationship between preference and eigengene expression, and the context‐dependent relationship between preference and eigengene expression indicated the interaction between preference and eigengene expression. Intensity of color indicates the significance of the relationship (adjusted *p*‐value). Each row represents the WGCNA module (color bars on the left match the named module on the far right). Three modules are significantly correlated with preference; however, none of the modules differed across social contexts or for the interaction between social context and preference.

While correlations between individual genes and behavior are a powerful approach to identifying candidate genes, we know that genes are non‐independent in expression and function. Identifying how networks of co‐expressed genes associated with behavior can be integrated with traditional individual gene‐level analyses to identify candidate genes robustly associated with behavior using distinct analysis approaches. We used weighted gene co‐expression analysis (WGCNA) to cluster genes based on their respective expression patterns into gene modules and summarize the co‐expression of genes at a module level rather than an individual level. In total, genes clustered into 25 co‐expression modules (excluding the gray module). The number of genes in each module (i.e., module size) ranged from 193 genes to 2504 genes (orange and turquoise modules, respectively; Figure 4). While none of the modules differed between social contexts or exhibited an interaction effect between context and preference, three modules associated with preference behavior (black: *F*_1,13_ = 10.9, *t* = 3.30, adjusted *p*‐value = .048; blue: *F*_1,13_ = 14.0, *t* = 3.74, adjusted *p*‐value = .035; green‐yellow: *F*_1,13_ = 13.5, *t* = −3.67, adjusted *p*‐value = .035) (Figure 4).

### 3.5 Genes robustly associate with mate preference identified by combining *limma* & WGCNA analyses

To examine the genes associated with preference based on both independent bioinformatic approaches (*limma* and WGCNA), we compared the ‘mate preference’ gene subset (Figure 2d) to those genes with expression highly correlated with WGCNA preference modules (i.e., module membership). Module membership (MM) measures the correlation between the expression of each individual gene and the module eigengene of each module (Young et al., 2023b: Tables S2 and S3). We found 415, 455, and 262 genes with high module membership (MM > |0.8|) with the black, blue, and green‐yellow modules, respectively.

**FIGURE 5:**
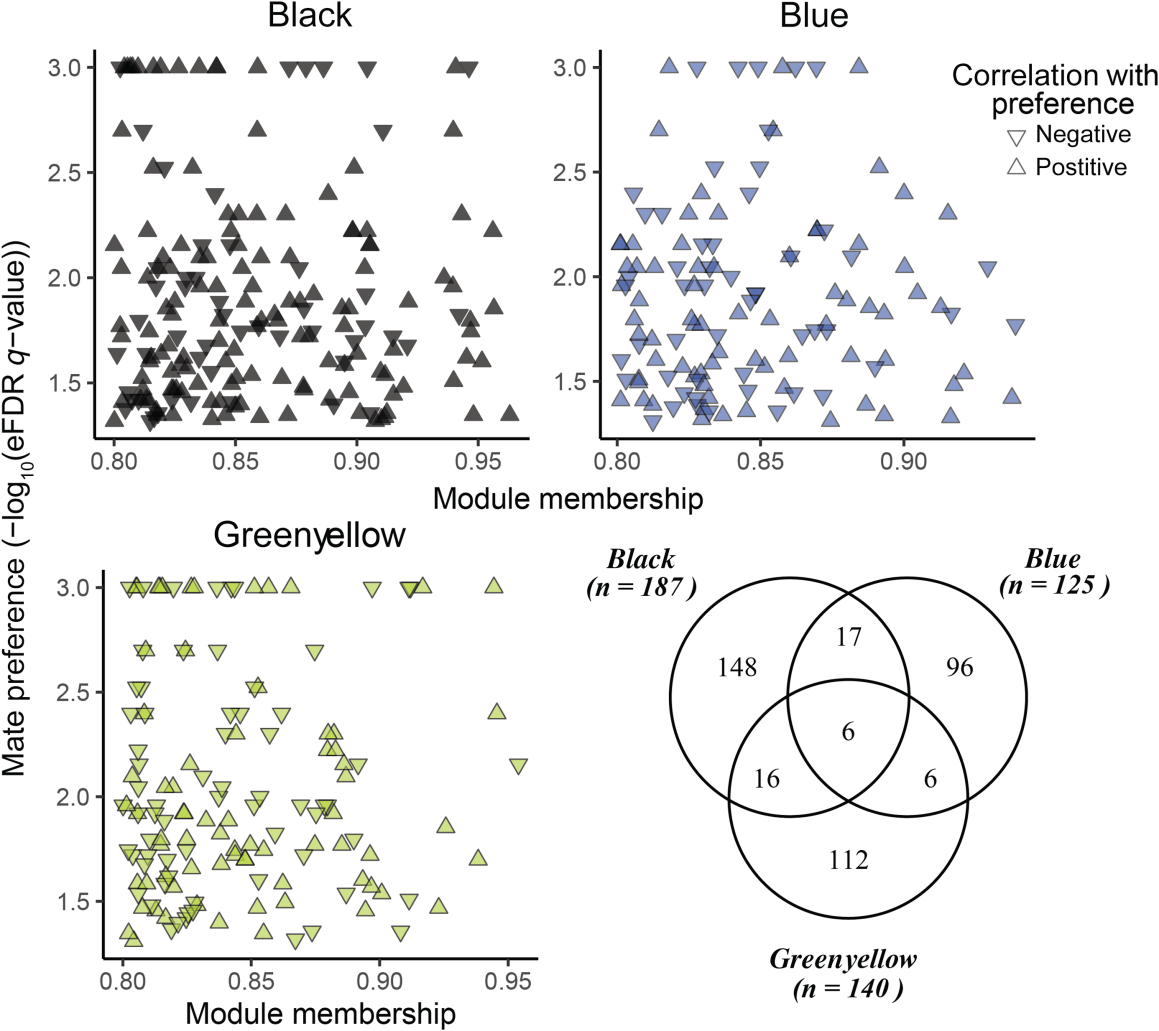
‘Refined mate preference genes’ were identified as genes with both strong correlation (i.e., module membership, MM > |0.8|) with significant preference modules (black, blue, and green‐yellow; Figure 4) and those with significant correlation with mate preference in the male‐interaction context (Figure 2d, blue). Upward and downward‐facing triangles indicate genes positively and negatively correlated with mate preference, respectively. Genes can have high module membership of more than one module, we identified the intersection among genes (shown in the Venn diagram). Combining the gene sets across the three modules identified 401 unique genes. Module membership and mate preference correlations for are provided in Tables S2 and S3 (Young et al., 2023b).

To generate a refined set of candidate genes robustly associated with mate preference, we identified genes significantly associated with preference in mating context (*limma*: *p*‐value < .05 and eFDR < 0.05; Figure 2) and strong association with preference modules (WGCNA: MM > |0.8|). This approach refined our ‘mate preference’ gene lists from 1665 *limma*‐identified (Figure 2c) to a total of 401 genes that are associated with mate preference behavior (Mm group: females interacting with male stimuli; Young et al., 2023b: Table S3). Of these genes, 187 were in the black module, 125 in the blue module, and 140 fell in the green‐yellow module; a small number of genes (*n* = 45) had high module membership in multiple modules (Figure 5). Genes having both a stong association with preference modules in WGCNA and high significance in *limma* may indicate genes that play a key role in preference behavior.

## 4 DISCUSSION

As the field of behavioral neurogenomics works towards a finer‐grained understanding of the relationship between brain gene expression and behavior, we have identified tools and techniques to help narrow the lens. For instance, the advent of single‐cell transcriptomics has allowed us to drastically improve our characterization of the spatial heterogeneity of neurons coordinating behavior. The use of time course transcriptomics (Bloch et al., 2018; Bukhari et al., 2017), careful selection of group controls (Cummings et al., 2008; Fischer et al., 2021), and individual‐level examinations of behavioral genomic responses (Whitfield et al., 2003) all provide higher resolution towards understanding the diverse brain response modulating specific behaviors. Here we incorporate some of these best practices (e.g., individual‐level examination) while introducing some novel refinements (a dual purpose non‐mating social and behavioral control) to advance our understanding of mate preference neurogenomics.

We identified sets of genes associated with social context and preference behavior using whole‐brain transcriptomic expression. Leveraging expression analysis (*limma*) with gene co‐expression analysis (WGCNA) enabled us to capture genes linking context‐dependent behavior to neural gene expression in two central components: (i) genes that are differentially expressed between social contexts, and (ii) genes that vary by preference behavior in both a shoaling partner (same sex) and a mate choice context. The core focus of our analysis was to identify genes associated specifically with preference, namely mate preference. While transcriptomics is not a substitute for identifying causal relationships between genes and behavior, we note that refining our ability to link brain gene expression with individual variation in behavior can set the stage for discoveries of novel genes and gene pathways critical to regulating social behaviors of interest. Further, we recognize changes in gene expression occur at the cellular level rather than at the whole brain level and that different regions of the brain contain different cell types. However, there is abundant literature showing that the neural transcriptome, even when coarsely sampled at the whole brain (as was done here) or in other coarse dissections, is closely tied with behavior (reviewed in Fischer et al., 2021; Toth, 2019; Toth & Robinson, 2007; Zayed & Robinson, 2012). Moreover, recent studies comparing single‐cell and bulk‐sampled transcriptomics reveal that a core gene expression network is shared across neural cell types and emerges across spatial scales (Crow & Gillis, 2018; Harris et al., 2021; Kelley et al., 2018). While this suggests that differences in the expression of bulk‐sequenced tissues reflect differences in cell type proportions, as different cell types may up‐ or down‐regulated the core expression network, the candidate genes and pathways discovered are likely to persist across spatial scales.

### 4.1 Genes expressed in social context & preference

Importantly, and not surprisingly given the polygenic nature of social behavior, we found an order of magnitude more genes associated with variation in behavior than differences in social context (e.g., interacting with males vs. interacting with females, Figure 2a). Sets of genes were positively or negatively associated with preference behavior across 15 female *Poecilia latipinna* individuals interacting with either male or female stimuli (Figure 2). Consistent with our interpretation that different gene sets are associated with preference and affiliation, the majority of mate preference genes (>85%) were discordantly expressed with affiliation (Figure 2c).

### 4.2 Genes associated with mate preference show concordance with candidate mate preference genes

By identifying genes uniquely associated with preference in a male‐interacting context, we were able to disentangle 1665 mate preference genes (Figure 2b,d, blue; Young et al., 2023b: Table S3). These genes were uniquely or differentially expressed when compared to shoaling partner preference genes (Figure 2d, red; Young et al., 2023b: Table S3) and genes associated with affiliative behavior in a male‐interacting context (Figure 2c). Within these mate preference gene sets, we found many genes previously implicated in poeciliid mate preference behavior that are involved in synaptic plasticity such as importin 4 (*ipo4*) in *X. nigrensis* (Cummings et al., 2008), and neuropeptide Y receptor (*npy8ar*) in *X. birchmanni* (Delclos et al., 2020). The focal female *P. latipinna* here expressed these exact ‐or functionally similar‐ genes in the mate preference context and here we find that these synaptic plasticity genes correlate positively with female *P. latipinna* mate preference behavior (Figure 2d(i)). We also found a suite of synaptic plasticity‐related genes (*s1pr1*, *serpine2*) correlating in the opposing direction with female mate preference behavior (Figure 2d(ii), Young et al., 2023b: Table S2), suggesting that the neuroplasticity pathways associated with mate preference behavior have complex interactions. In addition to identifying ‘mate preference’ genes associated with synaptic plasticity functions, we also found a significant number of these genes related to immune function (Figure 3), many of which were confirmed when we compared our *limma* and WGCNA results.

### 4.3 Gene co‐expression patterns and candidate mate preference genes

Our co‐expression network analysis identified three modules of genes significantly correlated with preference behavior across contexts indicating a robust neurotranscriptomic response to preference behavior (Figure 4). When we compare individual gene‐level analysis of expression with gene co‐expression analysis, we find some interesting overlap as well as divergence of results. Both approaches identify discrete sets of genes associated with preference. However, while our individual gene‐level analyses uncovered over 2000 genes associated with preference in at least one social context, our co‐expression approach identified no gene modules associated with the interaction between preference and social context (Figure 4). This distinction is likely due to the fundamental differences between these two approaches. Gene‐level analysis of expression (*limma*) allowed us to build linear models that use group assignment information to identify individual genes associated with preference behavior and compare these model outputs between the social contexts. Co‐expression network analysis (WGCNA) uses an unbiased approach (i.e., blind to experimental group information) to combine individual genes into groups (i.e., modules) of co‐expressed genes. The downstream analysis compares those module‐level metrics (module eigengenes) between contexts and behavioral measures. We generated a network of co‐expressed genes for females combined from both social contexts which may mask variation between the contexts and the resulting absence of context‐behavior interactions. Future work with larger sample sizes will be able to explore whether co‐expression networks of genes are assembled in context‐specific ways.

Combining these two independent analyses, we identified a robust and refined set of candidate genes underlying inter‐individual preference decisions in a mate‐choice context, i.e., mate‐choice genes. Of these 401 genes (Figure 5; Young et al., 2023b: Table S3) a few notable immune genes were discovered (i.e., *c1ql4b*, a homolog for the immune complement pathway gene *c1ql4*, and *ptprsa*) highlight a role for immune gene pathways in mate choice. *C1ql4b* was negatively associated with male‐interaction preference in our refined mate preference genes (e.g., Figure 2a(ii) set). Interestingly, this gene was also associated with mate preference in the optic tectum of female *P. reticulata* (named *zacrp4* in Table S1 in Bloch et al., 2018). In the brain, the complement component, C1q, plays a role in synapse elimination during development and diseases (e.g., neurodegenerative disorders) (Presumey et al., 2017; Stephan et al., 2012). Another immune‐related gene found in the refined mate preference gene set, *ptprsa*, was positively associated with mate preference. It is important to note that this gene, which is functionally similar to *neuroligin 3* that has been previously identified as a mate preference gene in *X. nigrensis* (Cummings et al., 2008; Wong & Cummings, 2014), acts as a crosslinking protein that assembles synapses (*ptprsa*; Takahashi et al., 2011; Takahashi & Craig, 2013).

### 4.4 Immune genes play a role in neuroplasticity, social behavior, mate preference

Many of the genes that covaried with mate preference can be classified as having both immune and neuroplasticity functions (Figure 3; Young et al., 2023b: Table S3). While these results may simply be a result of annotation bias in gene ontology terms, they may also highlight a potential role that ‘immune’ genes play in the brain with regard to learning (Herz et al., 2021). Mate choice is a learning‐dependent process in many vertebrates (Delclos et al., 2020; Kavaliers & Choleris, 2017; Rosenthal, 2017; Wong et al., 2011). Hence, the suites of mate preference genes identified here that are classified with dual immune and synaptic plasticity functions are potentially capturing the neuroimmune signaling pathways that alter synaptic inputs to facilitate learning (possibly through their function in cell remodeling and cell tagging, e.g., complement system). For instance, in addition to *ptprsa* mentioned above, we found mate preference genes with dual immune and neuroplasticity functions such as *ccr7* that is involved in immune processes and long‐term potentiation, long‐term depression, and adult neurogenesis (Williams et al., 2014). The gene s*1pr1* that was identified as correlating negatively with mate preference behavior is involved in immunity as well as playing a notable role in neuroplasticity by promoting adult hippocampal neurogenesis and improving context‐specific memory (Efstathopoulos et al., 2015). Many genes in the mate preference category are involved in immune surveillance, for example, by functioning in cell trafficking and presenting antigens to lymphocytes (B cells & T cells). Immune surveillance involves lymphocyte trafficking (*s1pr1*), and leukocyte trafficking (*ccr7*). Beyond immune and neuroplasticity‐related genes, we identified several interferon genes that correlated with mate preference behavior (e.g., *sting1*, and novel ‘interferon‐induced protein 44‐like’, an ‘interferon‐alpha/beta receptor 1b‐like’ and ‘interferon‐induced protein 44‐like’ genes). Interferons are a type of immune cell known as cytokines and have been implicated in many social behaviors (Monteiro et al., 2017), including social preference in mice, rats, and zebrafish (Filiano et al., 2016; Kirsten et al., 2018), social stress (Murgatroyd et al., 2016), learning, memory, and cognition (Brynskikh et al., 2008; Yirmiya & Goshen, 2011) and social preference (Kirsten et al., 2018).

While we posit that it is the neuroplasticity role of these neuroimmune genes that are actively engaged during a mate choice context to facilitate learning, we cannot rule out that some preliminary stage of an immune response is being triggered. In a mating context, immune activity may serve as an adaptive strategy that offers pre‐emptive protection (reviewed by Morrow & Innocenti, 2012) against physical damage (Crudgington & Siva‐Jothy, 2000; Kamimura, 2007; Řezáč, 2009; Stutt & Siva‐Jothy, 2001) and/or pathogen transmission (Kavaliers & Choleris, 2017; Zhong et al., 2013). This previous research identified a pre‐emptive response at a whole‐body level, whereas here we have identified this immune gene engagement in the brain. While the brain is unlikely to undergo a direct immune challenge during a mating encounter, the engagement of these neuro‐immune pathways may trigger a pre‐emptive protection response beyond the brain. Increased expression of immune‐related genes suggests an investment in immune defense in females from high sexual conflict systems (Bagchi et al., 2021; McGraw et al., 2004); and is largely unexplored beyond invertebrate systems. Poeciliidae may serve as an attractive vertebrate model to test hypotheses about the anticipatory immune response to mating within the CNS.

Interestingly, this pattern of neuro‐immune response genes during mating encounters is not unique to sailfin mollies. Similar findings have been found in other poeciliids (*X. birchmani*; Delclos et al., 2020) as well as male (Carney, 2007) and female *Drosophila melanogaster* (Lawniczak & Begun, 2004; McGraw et al., 2004). What is different between the present study and these previous studies is that prior research found the immune response occurred in a post‐mating context (*Drosophila* studies) or in the presence of olfactory cues only (*X. birchmanni*). Here, we are observing this neurogenomic response of immune‐related pathways before physical contact with a potential mating partner using visual stimuli only. Our data highlight the potential role that ‘immune’‐related genes play in the brain during social decision‐making during mate preference behavior. As learning is evident in mate preference behavior, we hypothesize that immune genes contribute to the neuroplasticity involved in learning and memory.

## AUTHOR CONTRIBUTIONS

**Rebecca L. Young:** Conceptualization (supporting); data curation (equal); formal analysis (lead); methodology (equal); software (lead); supervision (supporting); validation (lead); visualization (equal); writing – review and editing (equal). **Sarah M. Price:** Formal analysis (supporting); investigation (equal); methodology (equal); visualization (equal); writing – original draft (lead). **Molly Schumer:** Investigation (equal); methodology (equal); resources (supporting). **Silu Wang:** Conceptualization (supporting); investigation (equal); methodology (equal). **Molly E. Cummings:** Conceptualization (lead); data curation (equal); funding acquisition (lead); project administration (lead); resources (lead); supervision (lead); writing – original draft (supporting); writing – review and editing (equal).

### OPEN RESEARCH BADGES

This article has earned Open Data and Open Materials badges. Data and materials are available at https://doi.org/10.18738/T8/RHYJQ5 and https://doi.org/10.18738/T8/3SSYRK.

## Supporting information


Table S1

Table S2

Table S3

Table S4

## Data Availability

Raw and processed RNA sequencing reads are available at the Gene Expression Omnibus series accession GSE233523. All data and scripts used for behavioral and transcriptomic analyses and figure construction are publicly available on the Texas Data Repository (https://doi.org/10.18738/T8/RHYJQ5; Young et al., 2023a). Supplementary Tables are publicly available on the Texas Data Repository (https://doi.org/10.18738/T8/3SSYRK; Young et al., 2023b).
